# Experimental Tuberculosis in the Wistar Rat: A Model for Protective Immunity and Control of Infection

**DOI:** 10.1371/journal.pone.0018632

**Published:** 2011-04-12

**Authors:** Amit Singhal, El Moukhtar Aliouat, Maxime Hervé, Vanessa Mathys, Mehdi Kiass, Colette Creusy, Baptiste Delaire, Liana Tsenova, Laurence Fleurisse, Julie Bertout, Luis Camacho, Damian Foo, Hui Chien Tay, Jie Yee Siew, Warda Boukhouchi, Marta Romano, Barun Mathema, Véronique Dartois, Gilla Kaplan, Pablo Bifani

**Affiliations:** 1 Novartis Institute for Tropical Diseases, Singapore, Singapore; 2 Department of Parasitology-Mycology, Faculty of Biological and Pharmaceutical Sciences, University of Lille-Nord-de-France, Lille, France; 3 Biology and Diversity of Emergent Eukaryotic Pathogens (BDEEP)–Center for Infection and Immunity of Lille, Pasteur Institute of Lille, Inserm U1019, CNRS UMR 8204, University Lille-Nord-de-France, Lille, France; 4 Communicable and Infectious Diseases, Scientific Institute of Public Health, Brussels, Belgium; 5 Groupe Hospitalier de 1'Institut Catholique Lillois (GHICL), Hôpital Saint Vincent, Université Catholique de Lille, Lille, France; 6 Public Health Research Institute (PHRI), TB Center, University of Medicine and Dentistry of New Jersey, Newark, New Jersey, United States of America; 7 Institut Pasteur de Lille, Lille, France; Fundació Institut Germans Trias i Pujol; Universitat Autònoma de Barcelona CibeRES, Spain

## Abstract

**Background:**

Despite the availability of many animal models for tuberculosis (TB) research, there still exists a need for better understanding of the quiescent stage of disease observed in many humans. Here, we explored the use of the Wistar rat model for the study of protective immunity and control of *Mycobacterium tuberculosis* (Mtb) infection.

**Methodology/Principal Findings:**

The kinetics of bacillary growth, evaluated by the colony stimulating assay (CFU) and the extent of lung pathology in Mtb infected Wistar rats were dependent on the virulence of the strains and the size of the infecting inoculums. Bacillary growth control was associated with induction of T helper type 1 (Th1) activation, the magnitude of which was also Mtb strain and dose dependent. Histopathology analysis of the infected lungs demonstrated the formation of well organized granulomas comprising epithelioid cells, multinucleated giant cells and foamy macrophages surrounded by large numbers of lymphocytes. The late stage subclinical form of disease was reactivated by immunosuppression leading to increased lung CFU.

**Conclusion:**

The Wistar rat is a valuable model for better understanding host-pathogen interactions that result in control of Mtb infection and potentially establishment of latent TB. These properties together with the ease of manipulation, relatively low cost and well established use of rats in toxicology and pharmacokinetic analyses make the rat a good animal model for TB drug discovery.

## Introduction

The emergence of multidrug-resistant (MDR) Mtb strains and more recently, extensively drug-resistant (XDR) strains is a serious health care problem worldwide [1]–[4]. MDR strains are resistant to isoniazid (INH) and rifampicin (RIF). XDR strains are defined as resistant to fluoroquinolones and one of the three injectable drugs – capreomycin, amikacin and kanamycin, in addition to INH and RIF. The loss of use of these drugs in patients with MDR or XDR TB has highlighted the urgent need to develop new therapeutic interventions with novel targets, to successfully treat drug resistant TB. Furthermore, the majority of first and second line drugs that target growing bacilli are less efficient at killing non-replicating organisms. Therefore, the stop TB partnership and other funding organizations have stepped up efforts to develop new drugs that target both growing as well as non-replicating bacteria, thereby reducing the probability of development of latent disease.

Many of the advances in our understanding of *Mycobacterium tuberculosis* (Mtb) pathogenesis and host immunity to the infecting bacilli have been achieved through experimental research using various animal models. These animal models are also indispensable in the process of TB drug development. The mouse model, the most commonly used animal in TB research, gives rise to a chronic state in which infection in the lung stabilizes at a high level, steady state bacillary load. Mice lack some of the features characteristic of human TB disease including maturation of granulomas and the development of central necrosis, liquefaction and cavity formation [5]. Guinea pigs are highly susceptible to Mtb infection: a single bacillus can cause fatal disease within several months [6]. In these animals central necrosis is seen in the granulomas. Rabbits, historically considered to be resistant to Mtb infection, have recently been shown to develop chronic progressive granulomatous disease or control of infection depending on the virulence of the Mtb strains used for infection [7], [8]. The major advantage of this model is the resemblance of the histopathology of granuloma formation and maturation (necrosis, liquefaction and cavity formation) to human disease [9]. However, there is a paucity of commercially available immunologic reagents for the rabbit. The animal model that mimics most accurately the full spectrum of TB infection and disease in humans is the low dose pulmonary infection of Cynomologus macaques [10]. Solid, necrotic or cavitary granulomas are frequently observed within the same monkey. Further, about 50% of monkeys show clinical characteristics of latent TB [11]. Unfortunately, this model has the disadvantage of high cost and complex bio-containment considerations. These animal models, while addressing many aspects of human TB disease, lack a cheap and convenient model for studying latent infection and for testing new anti-TB drugs targeting non-replicating persistent Mtb.

Rats have been shown to be highly resistant to Mtb. Early studies suggested that infection with a high dose of Mtb could neither kill the rats nor produce typical TB lesions and tuberculin sensitivity [12], [13]. Later reports found that Mtb could disseminate to different organs in the rat and cause delayed type hypersensitivity responses to tuberculin, similar to observations in some strains of mice [14]–[16]. Lefford and colleagues showed that Mtb infected rats mount a rapid and specific immune response [17]. Recent studies on the rat have reported on the histopathology and bacillary load in the lungs of Mtb infected animals [18]–[20]. In the present study we explored the features of the Wistar rat model of Mtb infection and disease. Our results show that the containment of the disease and the characteristics of the host response to Mtb infection are both strain and dose dependent.

## Results

### The effect of Mtb strain on the response to infection in the lungs of Wistar rats

**Figure 1:**
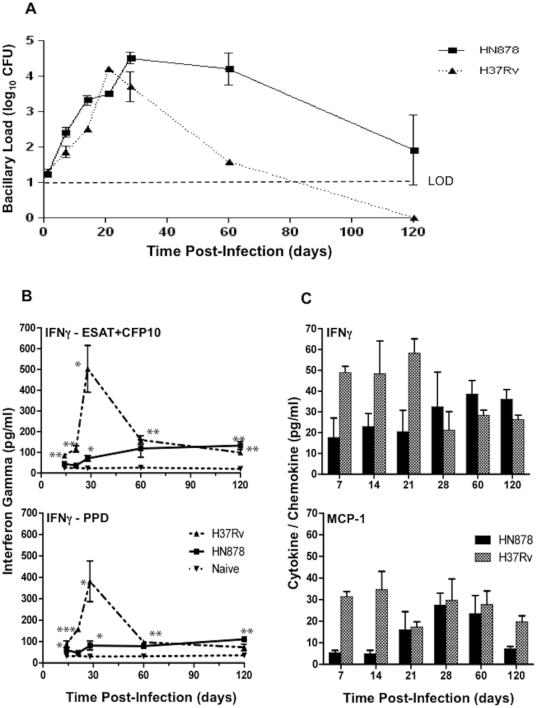
Bacillary load and extent of Th1 response in the lungs of Wistar rats infected by the endotracheal route with *Mycobacterium tuberculosis*. **A**, Number of Mtb colony-forming units in the lungs of rats infected with HN878 or H37Rv. The difference in Mtb load between the two strains at day 14 and 60 was significant (P<0.005). Values are means ± SD. **B**, Interferon gamma (IFN-γ) production by lung cells from Mtb infected rats following *ex vivo* stimulation with ESAT6+CFP10 and PPD antigens. Values are means ± SE. **C**, IFN-γ and monocyte chemotactic protein-1 (MCP-1) levels in the BAL fluid of rats infected with HN878 and H37Rv. Values are means ± SEs. (*P<0.05, ** P<0.01, *** P<0.001).

Six-week old female Wistar rats were infected with a low dose of the laboratory strain H37Rv or the clinical isolate HN878 and the bacillary load in the lung was evaluated over time. Similar growth of the bacilli was seen during the first three weeks of infection with either strain (Figure 1A). Thereafter, H37Rv bacillary counts dropped, below the level of detection (LOD) between 60 and 120 days post infection. In contrast, HN878 CFU numbers remained elevated in the lungs up to 60 days post infection, followed by a variable reduction in the numbers of organisms by 120 days. Similar low HN878 bacillary loads and H37Rv clearance were still seen at 240 days post infection (data not shown).

The differential efficacy of clearance of H37Rv versus HN878 was associated with different levels of IFN-γ production by cells isolated from the lungs and stimulated *ex vivo* with mycobacterial antigens. Up to 28 days post infection, cells isolated from H37Rv infected rat lungs produced increasing levels of IFN-γ when stimulated with ESAT-6+CFP-10 or PPD (Figure 1B). In contrast, cells isolated from the lungs of low dose HN878 infected rats produced consistently lower levels of the IFN-γ. By 60 and 120 days post infection, similar low levels of IFN-γ were produced by cells from the lungs of both groups of rats. When the BAL fluid of infected rats was evaluated for the presence of IFN-γ and MCP-1, a similar differential response was noted. Both IFN-γ and MCP-1 were present at higher concentrations at early time points in the BAL from H37Rv infected animals compared to HN878 infected animals (Figure 1C).

At this low dose of infection, no granulomas were formed in the lungs of H37Rv infected rats and very small (0.2 to 0.7 mm^2^) granulomas were observed in the lungs of HN878 infected rats (data not shown). Thus, the accelerated clearance of H37Rv from the lungs of infected rats was associated with significantly higher transient Th1 type cytokine production by mononuclear cells isolated from the infected lungs. These results suggested that similar to the mouse and rabbit, in the rat, HN878, a Beijing family clinical isolate of Mtb, is less immunogenic and therefore more virulent than H37Rv.

**Figure 2:**
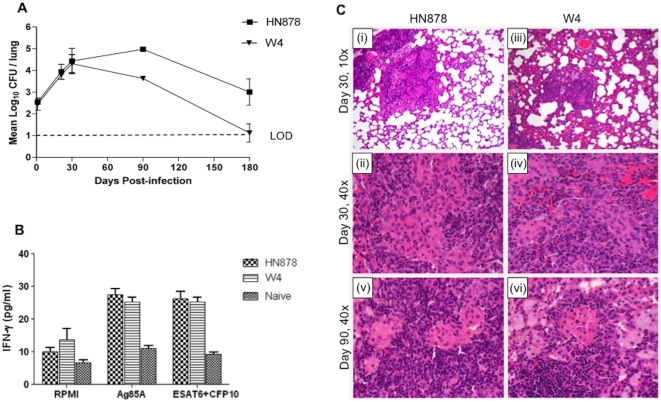
Response to infection of Wistar rats with two closely related Beijing Mtb strains. **A**, Pulmonary bacillary load in Wistar rats infected with HN878 or W4. Values are means ± SDs. The difference in Mtb load between both strains at day 90 (P = 0.001) and 180 (P = 0.019) was significant. **B**, Interferon gamma (IFN-γ) production by lung cells from Mtb infected rats following *ex vivo* stimulation with ESAT6+CFP10 or Ag85A antigens at day 30 post infection. Values are means ± SE. **C**, Histopathologic micrographs of lungs of rats infected with *M. tuberculosis* HN878 versus W4. (**i**) Infection with strain HN878, 30 days post infection, H&E, magnification 10×. Small, well organized granulomas, demarcated from the rest of the parenchyma. (**ii**) Same as (i) at magnification 40×. Distinct area rich in macrophages, surrounded by lymphocytes. (**iii**) Infection with strain W4, 30 days post infection, H&E, magnification 10×. Increased alveolar cell wall thickening is seen. (**iv**) Same as (iii) at magnification 40×. Granulomas consist of macrophages and lymphocytes. (**v**) Infection with HN878, 90 days post infection, H&E, magnification 40×. Well organized granulomas with areas rich in macrophages surrounded by large numbers of lymphocytes. (**vi**) Infection with W4, 90 days post infection, H&E, magnification 40×. Well organized granulomas with a higher number of foamy macrophages.

To further investigate the rat response to infection with Mtb Beijing strains, HN878 and W4 (another clinical isolate of the Beijing family) were used to infect the animals. When about 500 CFU were inoculated into the lungs of rats the two strains grew similarly for the first 30 days post infection (Figure 2A). Thereafter, somewhat higher numbers of CFU of HN878 persisted in the lungs of infected rats compared to W4. From 90 to 120 days, lung infection by both strains was partially controlled. At 180 days no CFU were detected in 4/12 and 7/12 HN878 and W4 infected rat lungs, respectively. When leukocytes were isolated from the lungs of infected rats at 30 days post infection and evaluated for IFN-γ production following *ex vivo* stimulation with mycobacterial antigens, similar levels of the cytokine were produced by cells from both groups of rats (Figure 2B).

In response to this dose of infection, granulomas were formed in the lungs of both W4 and HN878 infected rats (Figure 2C). At 30 days post infection, mixtures of epithelioid and foamy macrophages and lymphocytes were seen in the lung lesions of both infected groups. However, the pulmonary response to W4 infection included discrete granulomas surrounded by significant alveolar cell wall thickening which was much reduced in the lungs of HN878 infected rats. In conclusion, both Beijing strains induced similar levels of T cell activation as determined by IFN-γ production at 30 days. However, a reduced control of the HN878 infection with higher bacillary loads at the later time points was noted. These results suggest that even relatively small differences Mtb strains, not always well defined, can give rise to differential responses to pulmonary infection. Moreover, these results also suggest that the HN878 inocula size determines the extent of pathology seen in the lungs of infected animals (Figure 2 versus data described in the text for low dose HN878 infection).

### The effect of W4 inocula size on the response to infection in the lungs of Wistar rats

**Figure 3:**
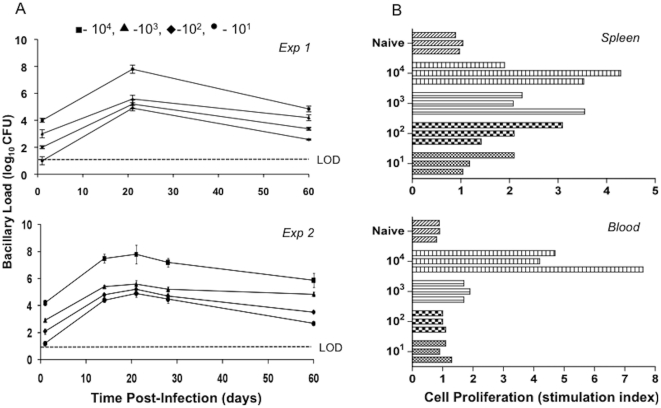
Bacillary load and extent of immune response in Wistar rats infected with different inocula of Mtb W4 strain. **A**, Number of Mtb colony-forming units in the lung of rats infected with different inocula of W4. Data from two different experiments are shown. Values are means ± SD. **B**, *In vitro* spleen and blood cell proliferation. Proliferation in response to ESAT6+CFP10 peptides was measured by [^3^H]thymidine incorporation. Each bar represents one animal.

To more carefully evaluate the effect of inocula size on the rat response to pulmonary Mtb infection, animals were inoculated with four different starting inocula, from 10^1^ to 10^4^ CFU of strain W4. The profiles of lung bacillary loads from two independent experiments are shown in Figure 3A. Bacillary growth was noted in response to infection with all inocula, peaking at 21 days post infection. Thereafter, a similar slow and steady decline in bacillary load was observed in the lungs of all four groups of rats by 60 days post infection. Enumeration of the bacillary load was also performed in different organs of the infected Wistar rats. No bacilli were recovered from the brain, blood, kidney and liver of the infected animals at any time points. However, sporadic bacterial dissemination to the spleen of infected animals was observed at 21 and 28 days. By 60 days post infection, no bacilli were found in any evaluated organs other than the lung (data not shown). Cells isolated from the spleen and blood of infected rats at 30 days post infection were evaluated for proliferation in response to *ex vivo* stimulation with ESAT-6+CFP-10. A clear inocula dose response was noted. Spleen and blood cells of animals infected with the highest dose gave rise to more extensive proliferation compared to lower dose inoculation (Figure 3B).

**Figure 4:**
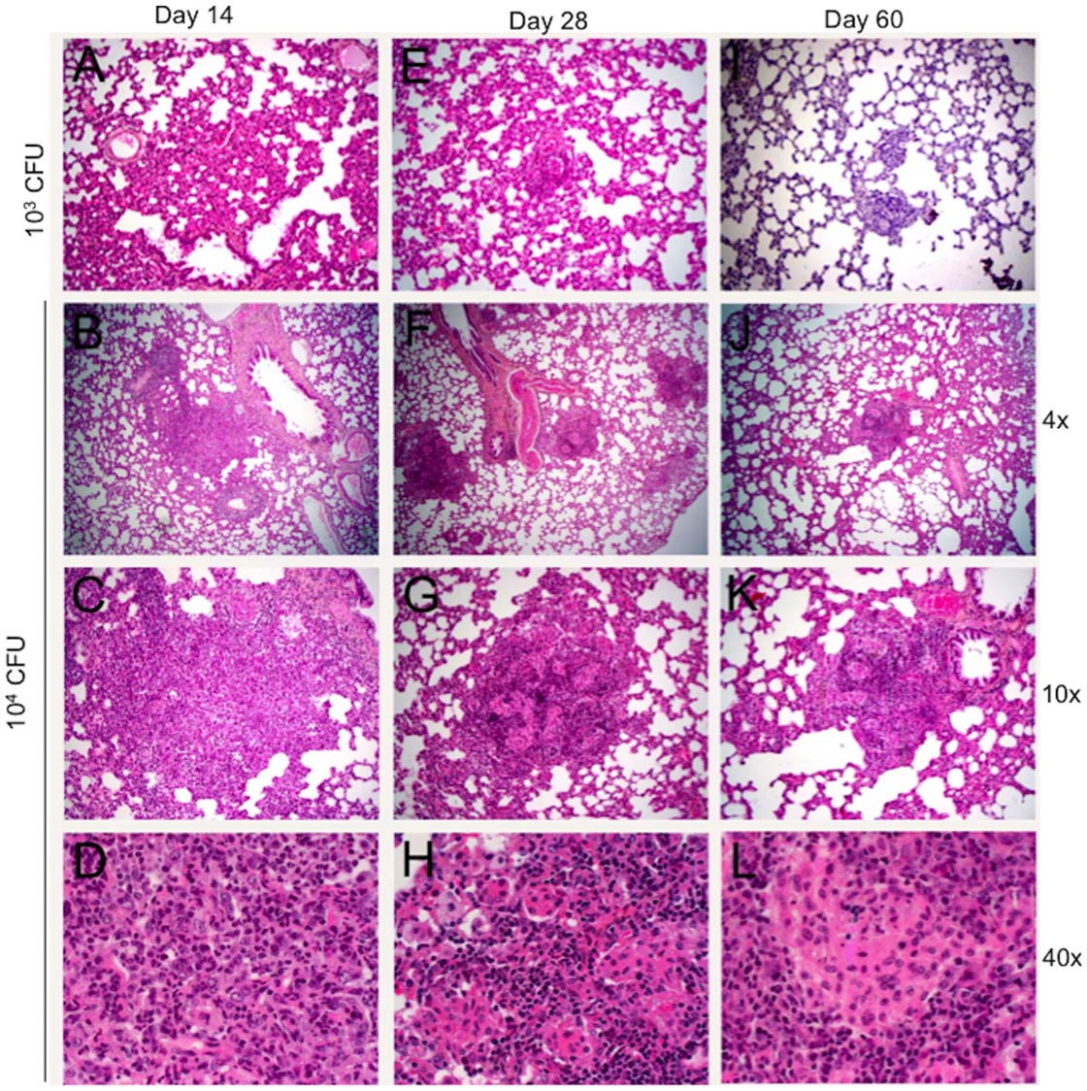
Histopathology of lungs of rats infected with *M. tuberculosis* W4, H&E. **A, E** and **I**, inoculum 10^3^ CFU, magnification 10×; **A**. Day 14 post infection. Alveolar cell wall thickening is observed. **E**. Day 28 post infection. Small cellular aggregates are seen. **I**. Day 60 post infection. Small granulomas have formed. **B, C** and **D** - inoculum 10^4^ CFU, 14 days post infection. **B**. Small peribronchial cellular aggregates are observed, magnification 4×. **C**. Magnification 10× and **D**, The lesions consist of mixed lymphocytes, PMNs and macrophages, magnification 40×. **F, G** and **H** - inoculum 10^4^ CFU, 28 days post infection; magnification 4×, 10× and 40× respectively. Small, well organized granulomas with central area of macrophages and cuff of lymphocytes. **J, K** and **L** - inoculum 10^4^ CFU, 60 days post infection; magnification 4×, 10× and 40× respectively. Well structured granulomas with differentiated macrophages.

Examination of the lungs of infected animals also showed a clear inocula dose response in terms of infection-induced pathology. The two lower doses of infection resulted in diffused cellular infiltration but no structured granuloma formation during the first 60 days of infection (data not shown). The intermediary inocula of 10^3^ gave rise to delayed formation of small (about 0.4 to 0.5 mm^2^) granulomas at 28 and 60 days post infection (Figure 4). The highest dose of infection gave rise to well organized small granulomas that on average increased in size from about 0.6 mm^2^ to 1.2 mm^2^ from day 14 to 28 post infection. At day 28, granulomas had central area of epithelioid macrophages and cuff of lymphocytes. By 60 days lesions were well structured with many lymphocytes, foamy macrophages and few multi-nucleated giant cells. AFB were detected in the lungs of both 10^4^ and 10^3^ infected rats (not shown). Necrosis and fibrosis were seldom seen. Taken together these results suggest that the magnitude of the host immune-pathologic response to Mtb W4 infection was dose dependent. However, the magnitude of this response did not determine the efficacy of clearance of the bacilli. Rather, CFU decreased similarly at all inocula, resulting in 60-day bacillary loads that were proportional to the initial inocula in each group.

### Histopathology appearance of lungs during subclinical infection

**Figure 5:**
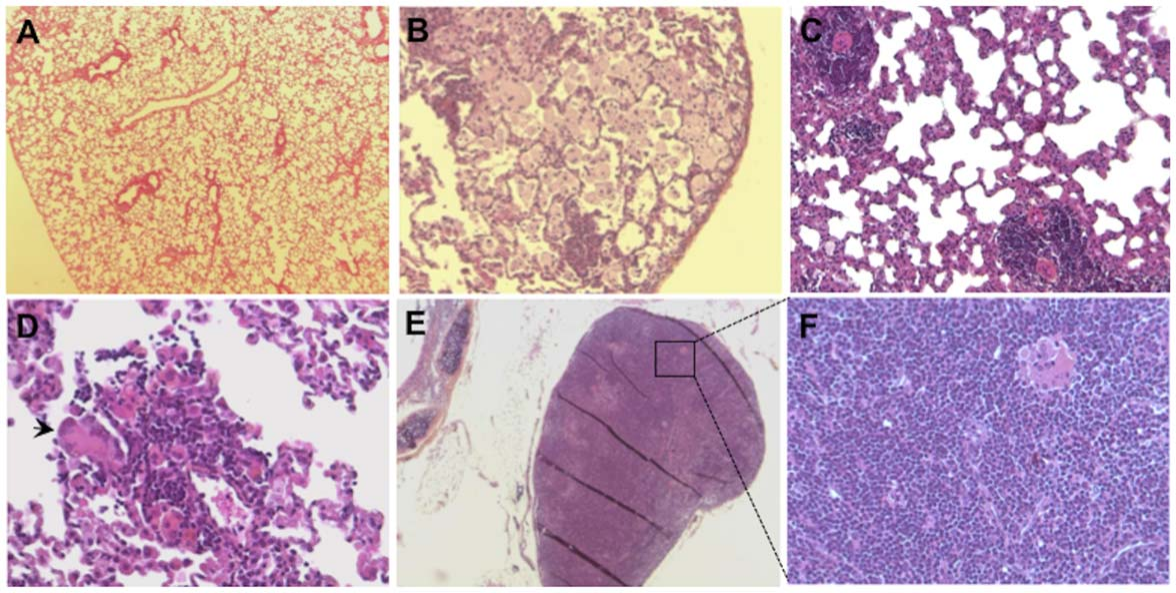
Histology of lungs from rats infected with W4 for 180 days. (A) Normal appearing lung parenchyma, magnification 6.5×. (B) Lung with variable numbers of foamy macrophages, magnification 10×. (C) Small granulomas containing lymphocytes and epithelioid cells, magnification 20×. (D) A small inflammatory cell aggregate containing a large numbers of lymphocytes and a multinucleated giant cell, magnification 40×. (E) An infiltrated mediastinal lymph node with lymphocytes and epithelioid histiocytes, magnification 6.5×. (F) Same as E, magnification 20×.

By 180 days post infection Mtb HN878 and W4 could only be detected in some of the animals (see text relevant to Figure 2A) suggesting a possible late subclinical phase of infection in these animals. Examination of the lungs from rats with subclinical infection revealed a spectrum of pathologic appearances ranging from healthy lung parenchyma (Figure 5A) to exceedingly cellular tissue. The lungs of some of the animals displayed multiple small granulomas (0.2 to 0.4 mm^2^ in size) intermixed with foamy macrophages (Figure 5B). Other better formed granulomas consisted of mixed lymphocytes, epithelioid cells and Langhans' type giant cells (Figure 5C and D). Pulmonary lymph nodes from these animals contained scattered epithelioid histiocytes and multinucleated giant cell (Figure 5E and F). No AFB and no calcification were observed in the lungs of these rats.

### Immunosuppression mediated reactivation of infection in Wistar rats

**Figure 6:**
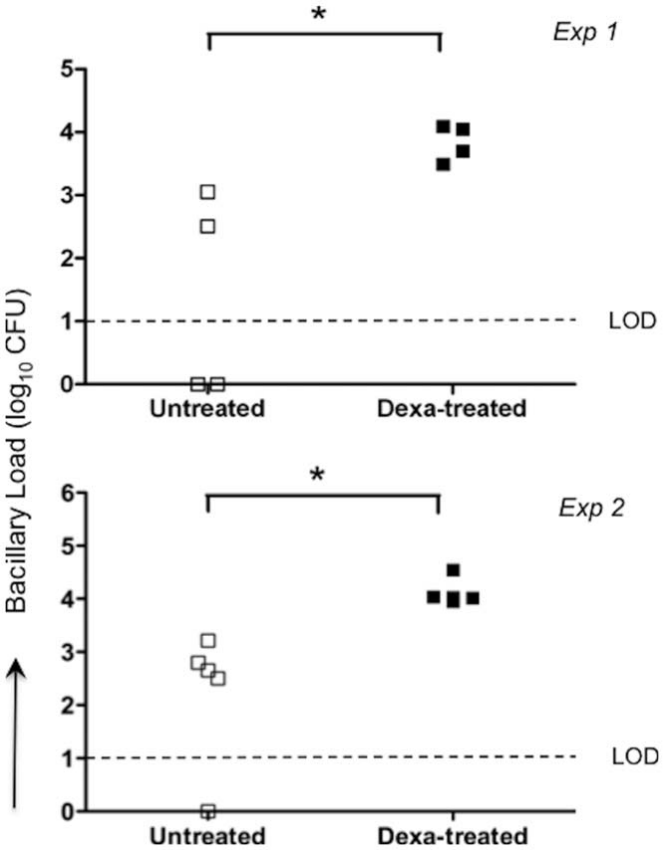
Bacillary load in the lungs of rats following immunosuppression. Rats were infected with 10^2^ CFU of *M. tuberculosis* strain W4 and either left untreated or treated with dexamethasone for one month starting at 100 days post- infection. Lung bacillary load was quantified at 130 days post-infection (* P = 0.025 and 0.036 for exp 1 and 2, respectively).

Wistar rats were infected with (100 cfu) Mtb W4 strain for 100 days. To induce reactivation of the bacilli, dexamethasone (2 mg/l) was given to some of the rats in drinking water from day 100 to day 130 post-infection. CFU were evaluated in the lungs of the 2 groups at 130 days. The dexamethasone treated rats had more cultivable bacilli compared to the untreated control animals, indicating a reduction in protective immunity facilitating the reactivation of bacillary growth (Figure 6).

## Discussion

An evaluation of the course of infection, host immunity and pathology in the lungs of Mtb-infected Wistar rats has identified some characteristics, which are different from other animal models of pulmonary TB. Upon low dose infection an increase in bacterial load associated with the induction of a strong adaptive immune response peaking by 28 days post infection was noted. Subsequent control of bacillary growth and thereafter suppression of growth in the lungs of infected rats was observed. Consequently, by 4 months post infection, depending upon the strain of Mtb used, the infection was brought under control and bacilli were eliminated, or bacillary loads were reduced significantly. This is different to what is observed in the mouse and the guinea pig but similar to what is observed in the majority of human hosts, where the infection is controlled and most individuals do not develop active disease but rather latent infection [21].

However, not much is known about the transition between infection in the lung and the establishment of the active or subclinical disease in humans. Do bacilli multiply first, leading to the development of an adaptive immune response which in turn controls and clears the multiplying bacilli and eventually gives rise to latent disease or is it the host innate immune response that plays an important role in clinical outcome? It has been reported that even in the Cynomologus macaque model, which is the representative animal model depicting human TB, the amount of gross disease seen at 60 days post-infection could not predict the establishment of latent disease [22].

Histopathology analysis of the lungs from rats infected with high inocula revealed several aspects of pathology, which are also similar to the human response but different from what is commonly observed in the mouse (Table S1) [23]. The formation of well organized granulomas containing multi-nucleated giant cells is a hallmark of human TB. In addition, resorption of lesions over time was noted at later time-points when bacilli were no longer detectable. In some animals foamy macrophages were frequent at the later time points. Foamy cells have been shown to be present during the resolution phase of pulmonary lesions [24] and have recently been proposed as the cells that might constitute a reservoir for long-term persistence of bacilli within the human host [25]. It is importatnt to note that in the present study all animals were infected by the endotracheal route and hence the lung lesions observed may differ from the pathology which develops following aerosol infection.

Differential virulence and immunogenicity of Mtb strains, defined here by the kinetics of bacillary growth and persistence in the lungs, and the extent of induction of IFN-γ production, has already been demonstrated in other animal models of TB. Using the rabbit model of tuberculous meningitis, it has been shown that the laboratory strain H37Rv is less virulent than the clinical isolates HN878 and W4 (W/Beijing genotype) [8]. The hypervirulence of HN878 appeared to be due to the failure of this strain to stimulate optimal Th1 type immunity in the mouse model [26]. This appeared to be associated with the production of phenolic glycolipid (PGL), which is not produced, by H37Rv [27]. Ordway *et al*. reported that the HN878 strain induces an initial potent Th1 response in mice followed by rapid down-regulation of this activity [28]. In the present study we noted that even relatively small differences among Mtb strains (as seen with the two Beijing isolates HN878 and W4) gave rise to different responses to the infection in the rat. Differential lung bacillary load has also been observed in rabbits infected with HN878 and W4 strains [8]. The interplay between the virulence of Mtb strain, control of infection and organization of the granulomas as reported in the present work is in agreement with a recent study, which describes how pathogenic mycobacteria exploit the granuloma as a mechanism for expansion and systemic dissemination [29].

It is difficult to study latency not only due to our lack of knowledge of the factors, both host and pathogen derived, which are responsible for the bacilli adopting a non-replicating state but also it is difficult to demonstrate the presence of latent bacilli in previously infected tissues [30]. In humans and Cynomologus macaques, latency has been described as absence of any clinical symptoms with a positive tuberculin skin test (TST) or T cell IFN-γ release assays in response to *in vitro* exposure to mycobacterial antigens [10], [31]. In the Cornell mouse model, latency has been defined as no detectable CFU following treatment with antibiotics. In the present study, we have observed reduction of CFU in the lungs of infected Wistar rats, to a point where bacilli were not detected by culture or were only observed at very low numbers. In this model, reactivation of infection upon immune suppression provides evidence for the presence of viable bacilli in the tissue even in animals where bacilli may not have been detected prior to immune suppression by the CFU assay [32]. Elwood *et al*. defined latency in cotton rats as the absence of granulomatous inflammation with positive lung bacillary cultures [33].

One of our aims was to benchmark the Wistar rat model as a tool to evaluate new anti-TB drugs. Drug efficacy experiments using the Wistar rat TB model have shown that the relationship between *in vivo* efficacy and area under the curve/minimal inhibitory concentration (AUC/MIC) follows the same trend in the rat as for other animal models and in patients (MH and VD, manuscript in preparation). Rats are inexpensive, simple to house and manipulate, with the added advantage of allowing repeated blood samplings. These properties favor the use of rats in statistically representative numbers. Moreover, Wistar rat are used as the primary species for ADME (Absorption, Distribution, Metabolism and Excretion) and toxicology studies in early drug development, with large comparison databases available in the public domain.

In summary, the Wistar rat offers several promising possibilities and additions as an animal model for TB. The kinetics of bacillary growth and control in the lungs, and the histopathology suggest that Wistar rats are more resistant to Mtb infection than the mouse. The model will allow us to understand the basic biology of Mtb from early control of infection to establishment of persistent disease, the mechanisms of drug diffusion in diverse host tissues, sterilization of non-replicating bacilli, analysis of drug tolerance and bacterial adaptability. It may therefore offer a good predictive and practical *in vivo* system for the screening of novel anti-TB drugs.

## Materials and Methods

### Ethics statement

All animals were maintained in accordance with protocols approved by the institutional animal ethical committee of ISP (Comité d'éthique concerté du CERVA, IPB at ISP). The authorization was approved by SPF Santé publique/FOD Volksgezondheid DG 4/Div 4: Bien-être animal et CITES - Dierenwelzijn en CITES (No LA2230389).

### Bacterial strains

Two well characterized clinical isolates of *M. tuberculosis* (Mtb), W/Beijing strains HN878 and W4 (TB Center, Public Health Research Institute, UMDNJ, Newark, NJ, USA) [25], [34], and the laboratory strain H37Rv were used in the experiments (ATCC25618). The strains were cultured in stationary Sauton medium in 500 ml cellular culture flasks to mid-log phase, washed, pelleted, re-suspended in phosphate-buffered saline (PBS), vortexed with 2 mm glass beads, left to settle and the supernatant was filtered through a 5 µm membrane. Subsequently the supernatant was transferred to a fresh tube for further washing/pelleting and finally adjusted in PBS/glycerol to an OD_600_ of 1.2 corresponding to approximately 2×10^8^ CFU/ml. The bacillary suspension was dispensed into aliquots and stored at −80°C. After one day an aliquot was thawed and bacilli were plated on 7H11 plates for CFU enumeration.

### Rats

Specific pathogen free (SPF) 6 week old outbred female Wistar Rats (Crl:WI) were purchased from Charles River, France. The animals were maintained in cages under biosafety-level-3 conditions at the Institute for Scientific Public Health (ISP), Brussels, Belgium. Throughout the experiments animals were fed sterile irradiated food and sterile water *ad libitum*. For immunosuppression rats were administered dexamethasone (2 mg/l) in drinking water [35].

### Endotracheal inoculation of rats with Mtb

Animals were anaesthetized by intraperitoneal injection with a cocktail consisting of ketamine: 75 mg/kg; diazepam: 1.2 mg/kg and atropine: 0.4 mg/kg and infected endotracheally as described [36]. Briefly, the inocula were deposited directly at the carina, the sternal angle of the bronchi, by passing a plastic needle through the mouth and trachea of the anaesthetized animal. This non-invasive technique has proven to be highly precise and consistent.

### Enumeration of mycobacteria in organs

At pre-determined time points post-infection rats were euthanized with sodium pentobarbital (55 mg/kg). One ml of blood was collected and diluted in PBS for colony forming unit (CFU) assay for each animal. In addition, the left lung or the whole lung, liver, kidneys, spleen and brain were all aseptically excised, washed in PBS and homogenized using a tissue homogenizer (Idea Works Laboratory Devices, Syracuse, NY). Bacterial counts in the organs of infected rats (n = 3–12 per treatment group) at each time point were determined by plating serial dilutions of tissue homogenates or blood on 7H11 agar plates and enumerating CFU after 3–4 weeks of incubation at 37°C.

### Histopathology and morphometric evaluation of granulomas

Segments of the lungs (3 animal/time point) were fixed with 10% neutral formalin, embedded in paraffin and processed for histology (N = 3/time point). Sections (5 µm) were stained with (i) hematoxylin-eosin-safran (HES), (ii) Sirius red and/or Volgens-Gomori (collagen types I and III for analysis of fibrosis), and (iii) Ziehl-Neelsen stain for acid-fast bacilli (AFB). Histologic sections were used for morphologic analysis of the size and number of granulomas at different time points post infection.

### Antigens

ESAT6+CFP10 fusion protein, PPD and recombinant antigen 85A (final concentration 5 µg/ml) were used for the lymphoproliferation assay and IFN-γ production. Concanavalin A and pokeweed mitogen (final concentration 4 µg/ml) were used as positive controls.

### Lymphoproliferation assay

The assay was performed as described previously [37]. Blood (1 ml) from each animal was collected into a heparinized tube and diluted with RPMI containing penicillin (100 U/ml), streptomycin (100 pg/ml), β-mercaptoethanol and 10% fetal calf serum (GIBCO). 200 µl of diluted blood was distributed in quadruplicates into 96-well round-bottom tissue culture plates (Falcon Labware) and incubated with various antigens for 5 days at 37°C in 5% CO_2_-95% air and 100% humidity. The spleen was homogenized and single cell suspensions (1×10^6^ cells) were cultured in a similar manner. On the evening of the 5^th^ day of incubation, 0.5 µCi of [^3^H] thymidine (New England Nuclear, Boston) was added to each well and the cells were further incubated overnight at 37°C in 5% CO_2_-95% air and 100% humidity. A multi-sample automated cell harvester (Skatron Inc., Sterling, Va.) was used to wash each well, and the effluent was passed through a filtermat (Skatron). [^3^H] thymidine incorporation into the cells was measured by liquid scintillation. Stimulation index was calculated as counts per minute (cpm) of cells stimulated with antigen divided by cpm of cells stimulated with RPMI alone.

### Cytokine/chemokine estimation in broncho-alveolar lavage (BAL) fluid

BAL was collected from the animals before excising the lungs from the chest cavity. Lungs were washed with 2.5 ml of 1x PBS to retrieve the BAL, the fluid was centrifuged and cytokines/chemokines were estimated in the BAL fluid using a multiplex flowcytomix kit (Bender MedSystems).

### Lung cell digestion

At the indicated time points following infection, rats were euthanized; lungs were aseptically removed from the pulmonary cavity, placed in RPMI-1640 medium and dissected. The dissected lung tissue was incubated with incomplete RPMI-1640 containing collagenase XI (1 mg/ml, Sigma) and type IV bovine pancreatic DNAse (30 µg/ml, Sigma) for 1 hr at 37°C under shaking condition. The digested lungs were disrupted by gently pushing the tissue through a cell strainer (100 µm, BD Biosciences). RBC's were lysed with RBC lyses buffer (Sigma), washed and lung cells were re-suspended in complete RPMI-1640 and total cell numbers were determined.

### *In vitro* interferon gamma (IFN-γ) measurement

Lung cells (1×10^6^ cells) were incubated with different antigens for 72 hr at 37°C in 5% CO_2_-95% air. After incubation, supernatants were frozen at −80°C until processed for IFN-γ measurement using an ELISA kit according to the manufacturer's instruction (eBioscience).

### Statistical analysis

All values were expressed as means ± SD or means ± SE. Two groups were compared using Student's *t*-test. Multiple groups were compared using one way ANOVA and Bonferroni's multiple comparison tests. All statistical analysis was carried out using GraphPad PRISM software. For statistical analysis a P value <0.05 was considered to be statistically significant.

## Supporting Information

Table S1 Comparison of TB pathology in Wistar rats with other animal models and human.
(DOC)
