# Supplementary material for: Experimental Tuberculosis in the Wistar Rat: A Model for Protective Immunity and Control of Infection
Source: PLoS One. 2011 Apr 12;6(4):e18632. doi: 10.1371/journal.pone.0018632 (PMC3075263; doi:10.1371/journal.pone.0018632)
Supplement: Table S1 — Comparison of TB pathology in Wistar rats with other animal models and human. (DOC) [file pone.0018632.s001.doc]

Table S1: Comparison of TB pathology in Wistar rats with other animal models and human.

| Model | Granuloma | Multi-nucleated giant cells | Necrosis | Cavitation | Hypoxia | Natural control | Susceptibility to Mtb | Use in Drug efficacy |
| --- | --- | --- | --- | --- | --- | --- | --- | --- |
| Mouse | Poorly organized | Absent | Rare^a^ | Absent | Absent | Absent | Moderate | Yes |
| Guinea pig | Well organized | Present | Present | Absent | Present | Absent | Very high | Yes^g^ |
| Rabbit | Well organized | Present | Present | Present^c^ | Present | Present^e^ | Variable | No |
| Monkey | Well organized | Present | Present | Present | Present | Present | Variable | No |
| **Wistar Rat** | **Organized** | **Present** | **Rare^b^** | **Absent** | **ND^d^** | **Present^f^** | **Low** | **Yes** |
| Human | Well organized | Present | Present | Present | Present | Present | Variable | Yes^h^ |

a - Only in C3HeB/Fej (lpr1-/-) hyper susceptible mice

b - Only in higher inoculum infected animals following immune suppression.

c - With some Mtb clinical isolates such as HN878

d - Not done, Study in progress

e - With some Mtb clinical isolates such as CDC1551

f - In low inoculum infection

g - Dose and Mtb strain dependent

h - Clinical trials
